# Transcriptome analysis reveals the long intergenic noncoding RNAs contributed to skeletal muscle differences between Yorkshire and Tibetan pig

**DOI:** 10.1038/s41598-021-82126-2

**Published:** 2021-01-29

**Authors:** Ziying Huang, Qianqian Li, Mengxun Li, Changchun Li

**Affiliations:** 1 Key Laboratory of Agricultural Animal Genetics, Breeding, and Reproduction of the Ministry of Education and Key Laboratory of Swine Genetics and Breeding of the Ministry of Agriculture, Huazhong Agricultural University, Wuhan, 430070 China; 2 Guangxi Yangxiang Co., Ltd. Production Center, Guigang, 537131 China

**Keywords:** RNA, Non-coding RNAs, Transcriptomics, Computational biology and bioinformatics, Molecular biology

## Abstract

The difference between the skeletal muscle growth rates of Western and domestic breeds is remarkable, but the potential regulatory mechanism involved is still unclear. Numerous studies have pointed out that long intergenic noncoding RNA (lincRNA) plays a key role in skeletal muscle development. This study used published Yorkshire (LW) and Tibetan pig (TP) transcriptome data to explore the possible role of lincRNA in the difference in skeletal muscle development between the two breeds. 138 differentially expressed lincRNAs (DELs) were obtained between the two breeds, and their potential target genes (PTGs) were predicted. The results of GO and KEGG analysis revealed that PTGs are involved in multiple biological processes and pathways related to muscle development. The quantitative trait loci (QTLs) of DELs were predicted, and the results showed that most QTLs are related to muscle development. Finally, we constructed a co-expression network between muscle development related PTGs (MDRPTGs) and their corresponding DELs on the basis of their expression levels. The expression of DELs was significantly correlated with the corresponding MDRPTGs. Also, multiple MDRPTGs are involved in the key regulatory pathway of muscle fiber hypertrophy, which is the IGF-1-AKT-mTOR pathway. In summary, multiple lincRNAs that may cause differences in skeletal muscle development between the two breeds were identified, and their possible regulatory roles were explored. The findings of this study may provide a valuable reference for further research on the role of lincRNA in skeletal muscle development.

## Introduction

Tibetan pigs, also known as ginseng pigs, have less subcutaneous fat, more lean meat, higher amounts of protein and amino acids, and better taste than Yorkshire pigs^1^. Tibetan pigs are particularly popular in the high-end market. The price of Tibetan pigs is at least five times the prices of other commercial pork varieties in the Chinese market. However, compared with traditional commercial pig breeds, such as Yorkshire, Tibetan pigs have slower growth rates and lower reproductive rates. At 12 months old, Tibetan pigs usually weigh approximately 25 kg^2^. As a typical Western breed, Yorkshire pigs have a high lean rate, rapid muscle growth, and heavy body weight^3^. Therefore, understanding the difference in skeletal muscle growth and development between the two breeds is beneficial to the genetic improvement of pigs in the future.

In pig breeding, the growth and development of skeletal muscles directly affect the quantity and quality of animal meat. Related studies have shown that skeletal muscle growth is affected by the number, size, and type of muscle fibers^4^. and the number and size of muscle fibers are closely related to the tenderness of pork^5,6^.

Long intergenic non-coding RNA (lincRNA) is defined as RNA transcripts longer than 200 nucleotides^7^. Many studies have revealed the potential effects of lincRNA between different pig species on skeletal muscle development. Zou et al^8^. found that multiple lincRNAs in Yorkshire pigs and Wunahua pigs may cause differences in growth and meat quality between the two breeds in unknown ways. Liang Zhou et al^9^. found that Linc-YY1 can promote myogenic differentiation and muscle regeneration by interacting with the transcription factor YY1. However, the function of most lincRNAs in muscle is still unclear. This study focuses on key lincRNAs and explores its possible effects on pig skeletal muscle development by analyzing the regulatory pathways it may participate in.

Earlier studies obtained transcriptome expression profiles of Tibetan pigs and Yorkshire through RNA-seq data. Among them, 209 genes were screened in TP, including multiple genes related to the formation of muscle fibers. The author speculates that it may play an important role in determining the growth rate and potential weight of pigs after birth^10^. In this study, we used this set of RNA-seq data to explore the potential role of lincRNA on porcine skeletal muscle development. DELs were obtained through pipeline analysis and differential expression analysis, and potential target genes in cis were predicted. A co-expression network of MDRPTG and its corresponding long intergenic non-coding RNA (lincRNA) was constructed and used to explore the potential role of DEL in the process of muscle fiber hypertrophy.

## Result

### Transcriptome assembly and lincRNA identification

**Figure 1:**
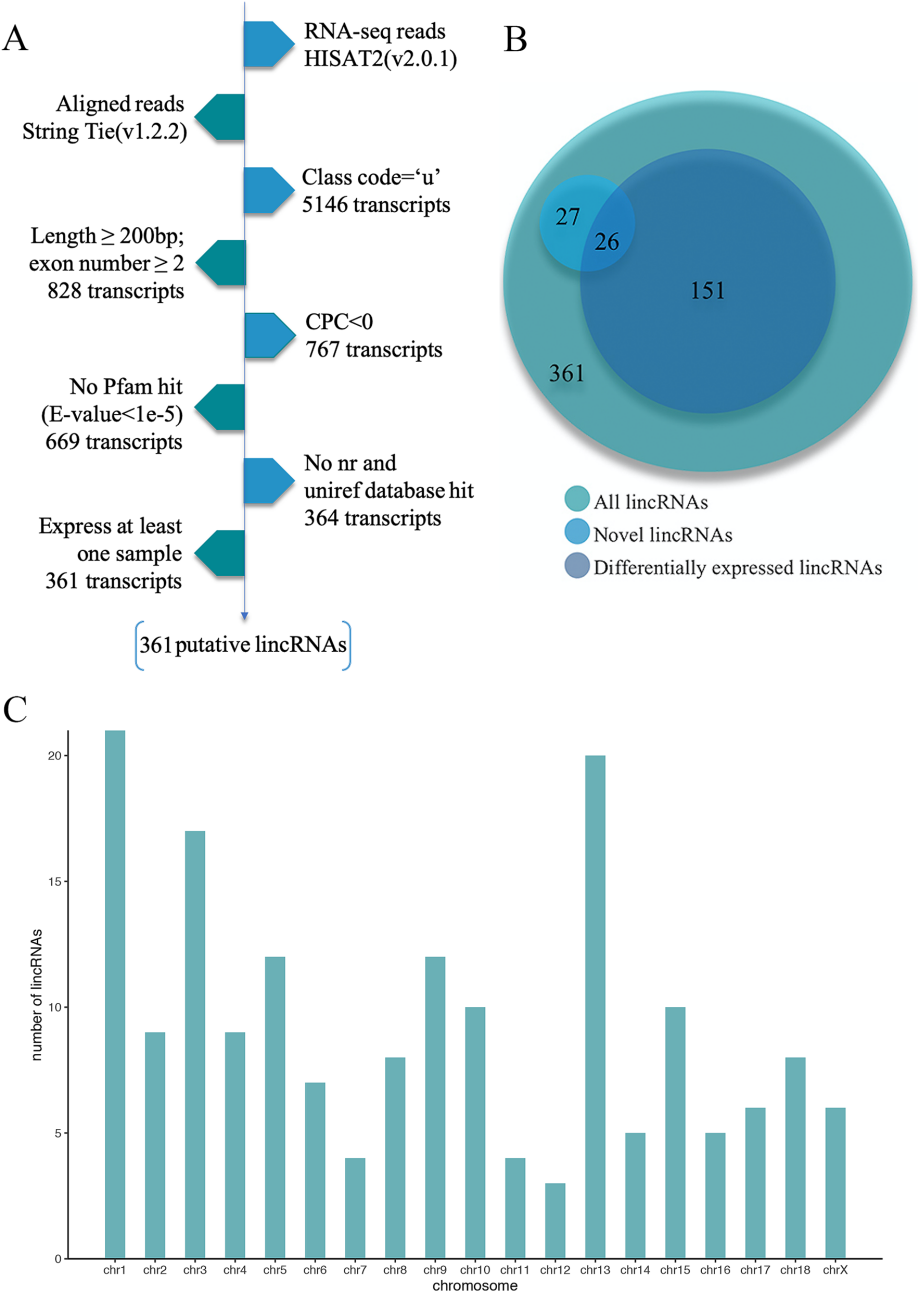
LincRNA analysis pipeline, classification and chromosome distribution. (**A**) LincRNAs identification pipeline. (**B**) Venn diagram of the proportion of different lincRNAs. (**C**) LincRNAs chromosome distribution.

Six published RNA-seq data (Fig. S1,Table 1) sets of two pig breeds (Tibetan and Yorkshire)^10^ were used in identifying lincRNAs that may cause phenotypic differences between the breeds. Potential lincRNAs were obtained on the basis of this pipeline(Fig. 1A). Approximately 245.05 of 258.43 million reads were mapped to the pig reference genome (Sus scrofa 11.1) by Hisat2. The mapped reads of each data were assembled into one set of transcripts with StringTie, and all of the transcripts from six data were merged into a nonredundant transcript set. A total of 828 transcripts, which were > 200 bp intergenic transcripts with more than two exons, were obtained from this pipeline. Finally, three different methods namely, Cpc, Hmmer, and Blast were used for the assessment of the coding potential of the transcripts. A total of 361 potential lincRNAs were obtained. In addition, 53 of the potential lincRNAs had no overlaps with currently annotated coding or noncoding transcripts (Fig. 1B). These lincRNAs are distributed in all chromosomes except the Y chromosome (Fig. 1C).

**Table 1:** The summary of data from RNA-seq for Tibetan (TP) and Yorkshire (LW) pigs.

Sample	Breed	Accession number	Clean reads	Mapping ratio (%)
TP_1	Tibetan pig	SRR5651381	40363474	94.69
TP_2	Tibetan pig	SRR5651382	49561190	94.09
TP_3	Tibetan pig	SRR5651383	41090604	94.77
LW_1	Yorkshire pig	SRR5651387	38321268	95.07
LW_2	Yorkshire pig	SRR5651388	44401474	95.25
LW_3	Yorkshire pig	SRR5651389	44689528	95.08

### Characterization of pig lincRNAs

**Figure 2:**
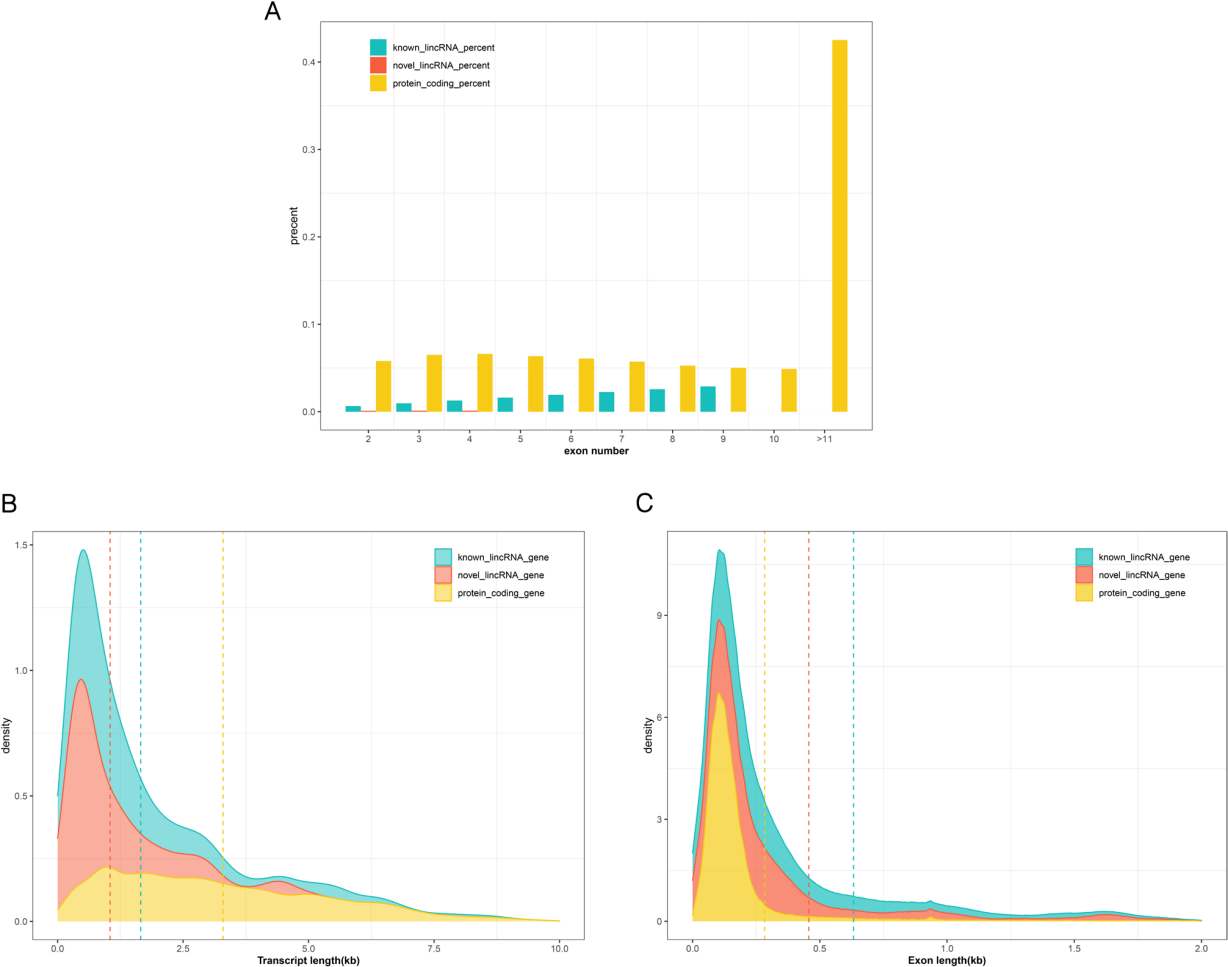
Characterization of identified lincRNAs. Characterization of identified lincRNAs. (**A**) The number of exons of different lincRNAs and protein-coding genes. (**B**) Probability density graph of the transcription length of different lincRNA and protein coding genes. (**C**) Probability density graph of the exon length of different lincRNAs and protein-encoding genes.

Previous studies have shown by comparing structural features that pig lincRNA is identical to the lincRNAs of other mammals (human and mouse)^11,12^. The pig lincRNA has fewer and longer exons than the coding gene; the lincRNA transcript, owing to their small number of exons, is shorter than the coding gene^13^. Thus, the present study compared the difference in exon number (Fig. 2A), length (Fig. 2B), and length of transcription (Fig. 2C) between lincRNA (known lincRNAs and novel lincRNAs) and protein coding genes in this data, consistent with previous reports^14^. The accuracy of lincRNAs obtained from the pipeline of this study was confirmed.

### Expression analysis of lincRNAs

**Figure 3:**
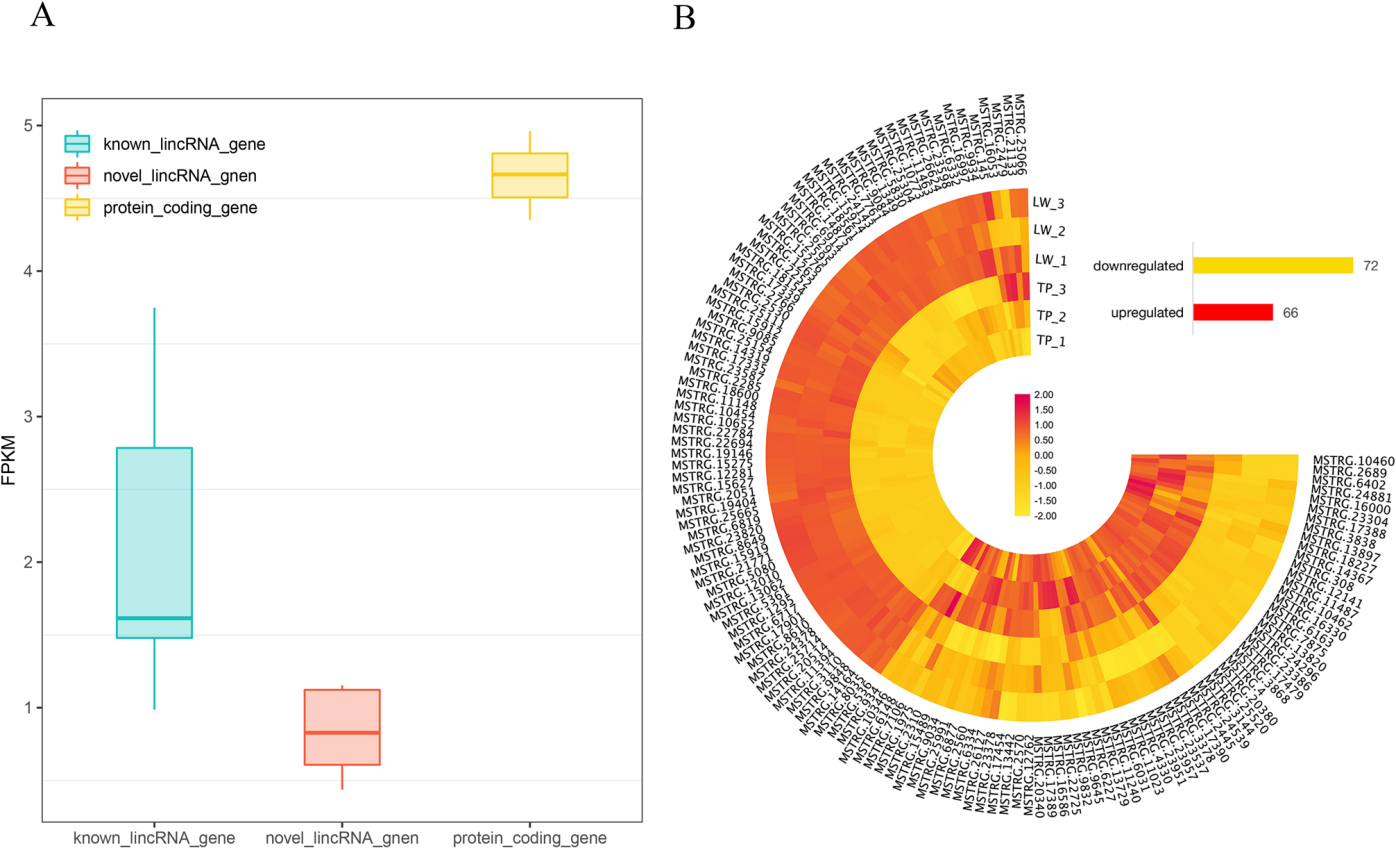
The results of expression analysis. (**A**) Expression levels of different lincRNA and protein-encoding genes. (**B**) Differential expression analysis of differential lincRNAs, the bar code represents the color scale of the log_10_^(FPKM)^ .

The mammalian genome is universally transcribes and encodes thousands of lincRNAs distributed throughout the genome, which are less conserved and have low expression levels^15,16^. The present study compared the average expression levels of 361 lincRNAs (known lincRNAs and novel lincRNAs) and protein coding genes from six samples to investigate whether this expression pattern is also present in pigs. The results showed that the average expression level of lincRNAs (known lincRNAs and novel lincRNAs) in pigs is generally lower than that of genes encoding proteins (Fig. 3A). In order to study the lincRNA that may cause phenotypic differences between the two breeds of pigs (Yorkshire pigs and Tibetan pigs). Deseq2 in the R was used to perform differential expression analysis on the two breeds of pig samples on the basis of expression levels. Between the two breeds, 66 of the 138 DELs of Yorkshire pigs were upregulated and 72 were downregulated (Fig. 3B). Between the two pig breeds, 326 of the 682 differentially expressed coding genes identified were downregulated and 356 were upregulated (Fig. S2).

### DELs target genes prediction and GO and KEGG enrichment analysis

**Figure 4:**
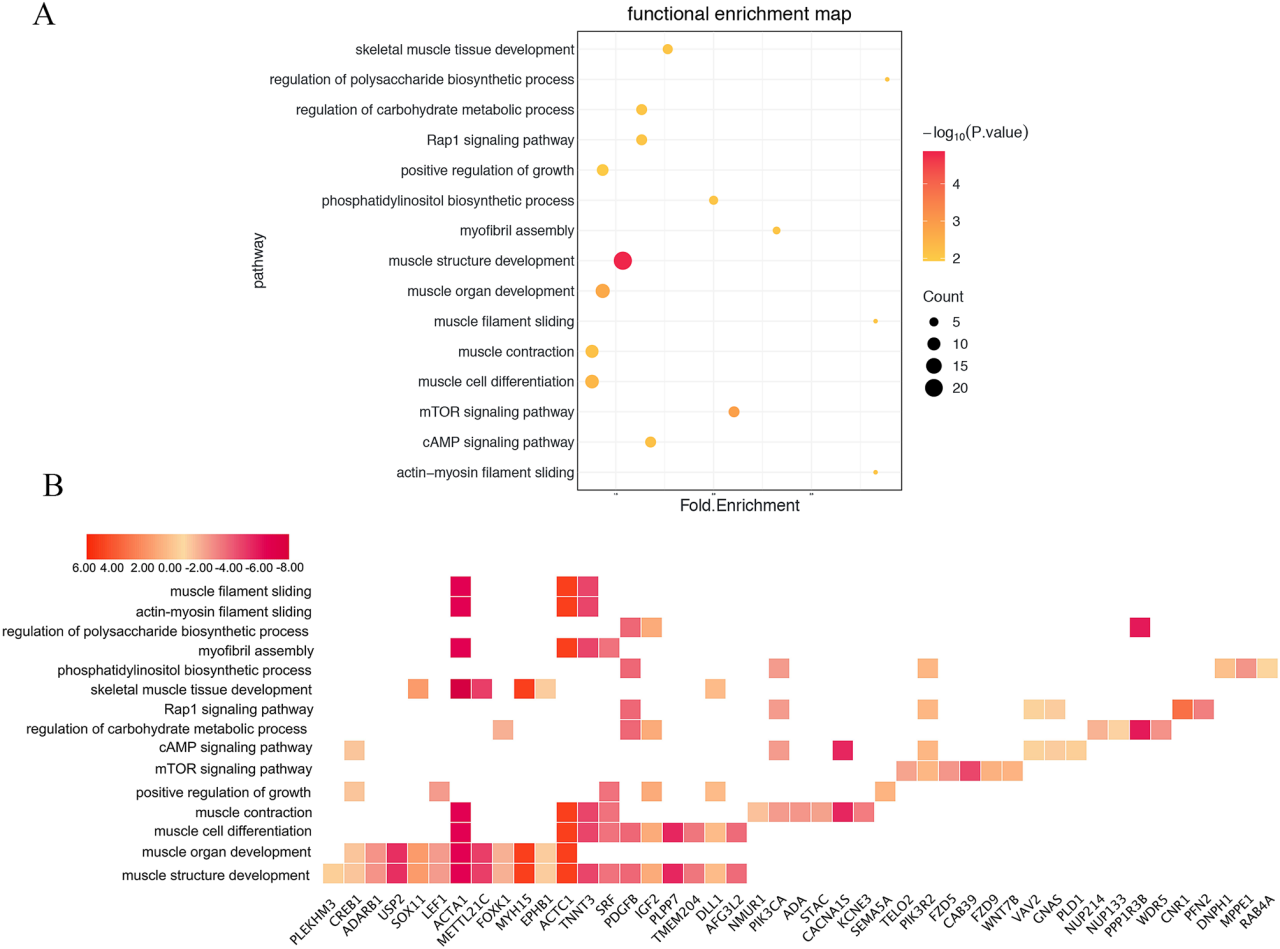
The results of Gene ontology and pathway analysis. (**A**) Gene ontology and pathway related to muscle development. (**B**) Gene expression in Gene ontology and pathway related to muscle development, the bar code represents the color scale of the log_10_^(FC)^.

Given that lncRNA can silence or activate cis-gene expression, it can act on neighboring genes at lncRNA sites^17^.Target genes (Table S1, the methods of target genes prediction refer to methods) in the range of 100 kb upstream and downstream of the DELs position were searched. Conducting the online GO and KEGG analysis^18–20^ (Table S2) through Metascape to explore the functions of target genes that may be regulated by DELs^21^. The results showed that 409 PTGs were significantly (*P* < 0.05) involved in 155 biological processes and 21 pathways. Many biological processes and pathways involved in muscle development (Fig. 4A). In addition, we found that the genes in the pathways related to muscle development differently expressed between the two species (Fig. 4B). It is speculated that the differential expression of target genes may be related to the differential expression of lincRNA between the two species.

### QTLs analysis of DELs and functional prediction

**Figure 5:**
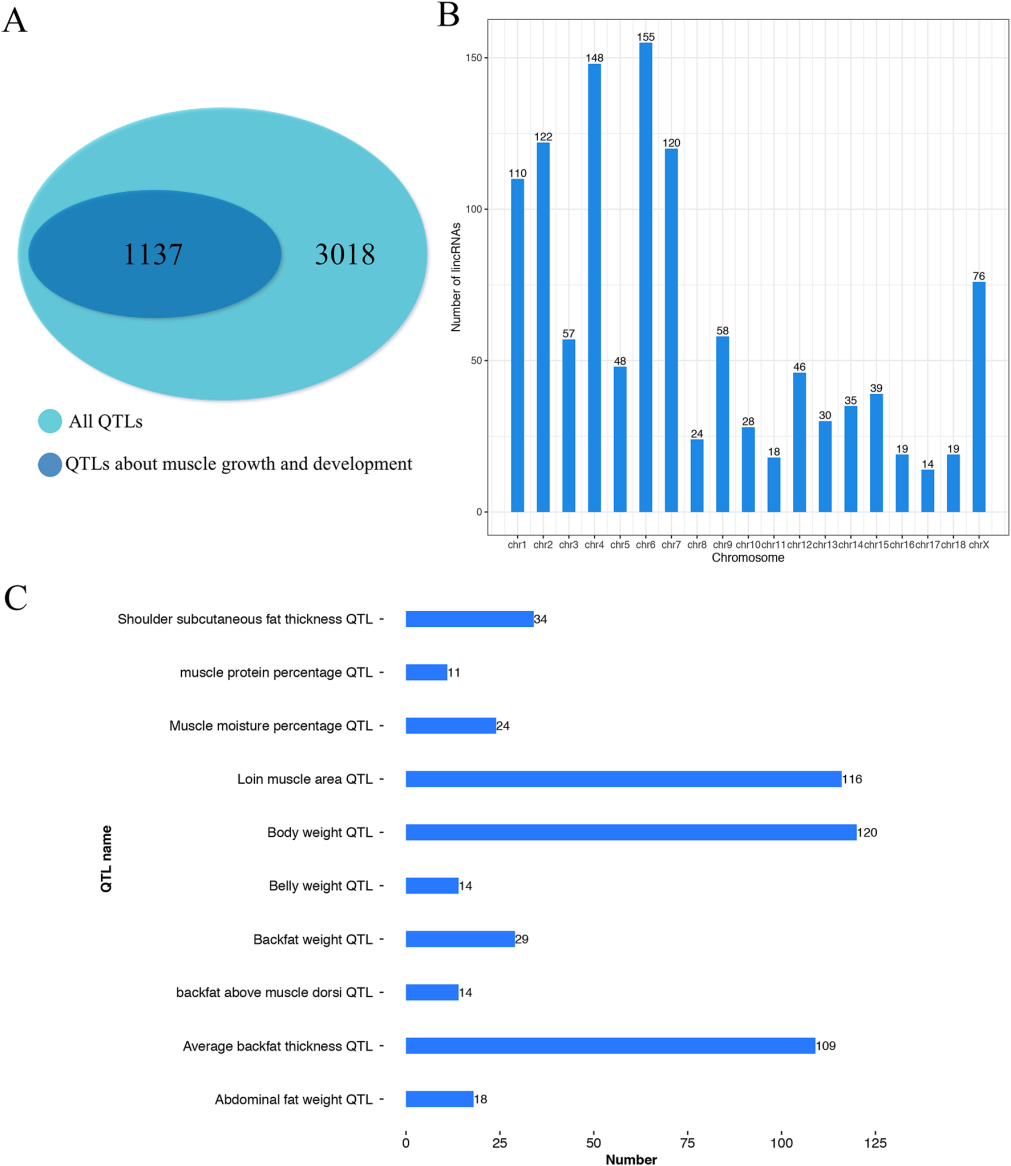
QTL analysis results. (**A**) Percentage of muscle development-related QTLs among all QTLs. (**B**) Distribution of QTL in each chromosome. (**C**) The Top 10 of QTLs.

The functions of DELs were further explored by performing QTL mapping analysis (Table S3) after the prediction of the target genes of DELs. The results indicated that approximately 37% of QTLs are associated with muscle growth and development (Fig. 5A). We calculated the chromosome distribution of QTLs associated with muscle development were mainly distributed on chromosomes 4 and 6 (Fig. 5B). Interestingly, the top 10 QTLs were associated with muscle development, and were mainly concentrated on the average back fat thickness QTL, waist muscle area QTL and body weight QTL (Fig. 5C). This result speculates that the potential function of DELs may be related to muscle growth and development.

### Construction of co-expression networks

**Figure 6:**
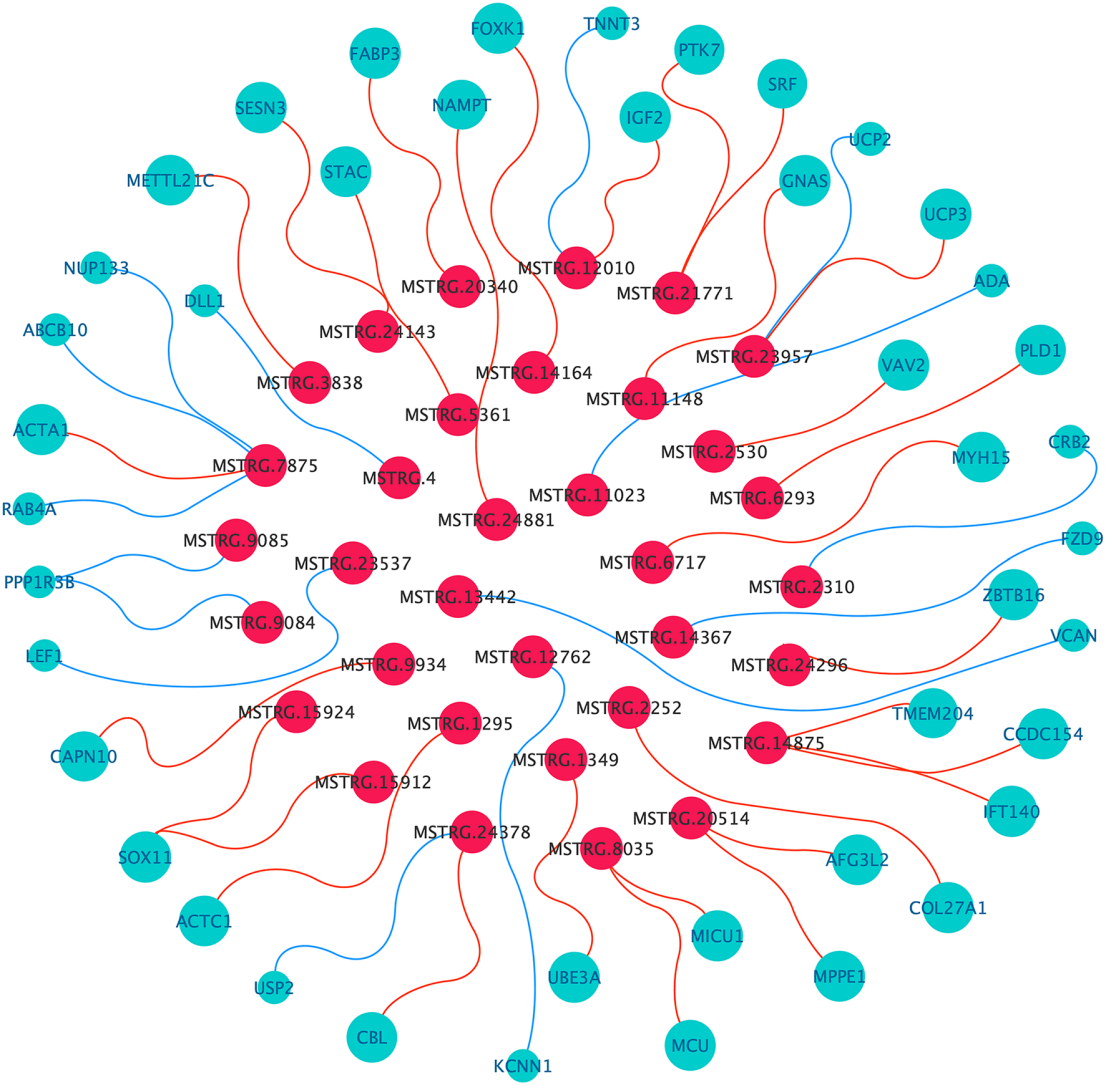
DELs-MDRPTGs co-expression network diagram. Green dots represent MDRPTG, red dots represent DEL; green lines indicate that DEL is negatively correlated with the corresponding MDRPTG, and red indicates that DEL is positively correlated with the corresponding MDRPTG.

To further explore the potential role of DELs in muscle development. We collected PTGs from biological processes and pathways involved in muscle development (Table S4). Such as the second messenger adenosine 3′, 5′-adenosine monophosphate (cAMP) pathway, which can affect the size of muscle fibers for a long time. Continuous activation of the cAMP pathway can lead to a pronounced hypertrophic response in skeletal muscle fibers^22,23^. And can promote animal hypertrophy, including muscular dystrophy, age-related atrophy and other animal models of various diseases against atrophy^24,25^. cAMP is also involved in muscle development and regeneration mediated by muscle precursor cells^26,27^. On the other hand, our predicted pathway also includes the core pathway IGF1–Akt–mTOR pathway that affects skeletal muscle hypertrophy^28^. Muscle hypertrophy is caused by increased protein synthesis and decreased protein degradation^29^. This pathway plays an important role in protein accumulation^30^.Based on the expression levels of these PTGs and the corresponding lincRNA, we construction the DELs- MDRPTGs co-expression network by using Cytoscape_3.6.1^31^ (Fig. 6). It was found that 34 of the 138 DELs may regulate PTGs which associated with muscle development, and we found that 25 of 34 DELs upregulate their target genes. In addition, there are eight DELs corresponding to more than two MDRPTGs, two MDRPTGs correspond to multiple DELs. Therefore, we speculate that there are some regulatory mechanisms related to muscle development between MDRPTG and DEL.

### Correlation verification between DEL genes and their PTGs

**Figure 7:**
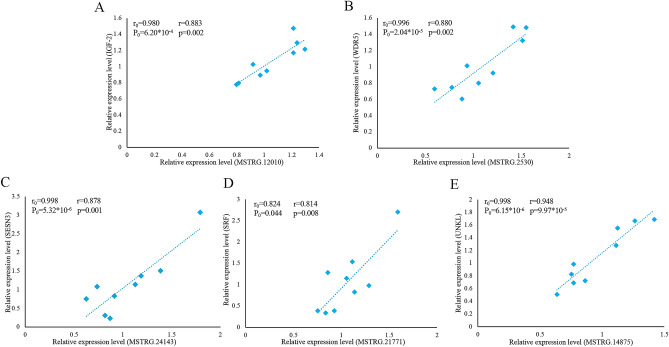
Linear regression of lincRNA and PTG expression. The r_0_ and p_0_ represent the Pearson correlation coefficient and p value of each pair of lincRNA and PTG in 6 samples, respectively; The r and p represent verification in 9 samples. (**A**) MSTRG.12010 vs IGF-2. (**B**) MSTRG.2530 vs WDR5. (**C**) MSTRG.24143 vs SESN3. (**D**) MSTRG.21771 vs SRF. (**E**) MSTRG.14875 vs UNKL.

In the PTG prediction section, we predicted 409 PTGs corresponding to 138 DELs. To confirm this result, we randomly selected 5 lincRNA genes with significant positive correlation based on their expression levels. The correlation coefficients were all greater than 0.80, and the *p* values were less than 0.05. We performed RT-qPCR experiments on nine samples, and the results were analyzed using linear regression. The expression levels suggested that the five pairs of lincRNA genes and their PTGs are significantly positively correlated, with a correlation coefficient greater than 0.80 and *p* value of less than 0.05. The experimental results of RT-qPCR showed that the results of the two datasets are in good agreement, further improving the reliability of the present study (Fig. 7).

## Discussion

The skeletal muscle is the largest organ in mammalian animals. In pigs, skeletal muscle have important economic significance for production, and understanding the development of skeletal muscle is important for improving productivity and meat quality. In this study, we identified 361 potential lincRNAs based on the designed pipeline and found that 53 of them are novel lincRNAs. At the same time, we obtained 138 DELs. LincRNA generally indirectly exerts its potential regulatory effect by regulating the target protein-coding genes^32^. Therefore, we predicted the PTG of DEL and the potential function of these PTGs. We found that multiple biological processes of these PTGs are related to skeletal muscle development. Such as muscle structure development, muscle organ development, muscle cell differentiation and skeletal muscle tissue development. Further, We also found that there are multiple PTGs involved in cAMP signaling pathway, which can promote muscle growth and protein synthesis^33^, and play a key role in embryonic muscle growth and development^34^. In addition, in muscle tissue, there is a balance between muscle synthesis and degradation, and rapamycin (mTOR) signaling plays a key role in regulating protein synthesis^35^. We found that multiple PTGs are related to mTOR signaling pathway. On the other hand, QTL mapping analysis of DELs showed that 1137 of the 3018 QTLs were related to skeletal muscle development. The proportion of loin muscle area QTL and body weight QTL is the highest.

Finally, to further explore the potential regulatory role of DEL on muscle development. We generated the MDRPTG-DEL co-expression network and studied PTG related to muscle development. The growth of skeletal muscle depends on muscle fiber hypertrophy, and the size of muscle fibers is increased when the rate of protein synthesis is higher than the rate of degradation. Under normal physiological conditions, the IGF-1-Akt-mTOR pathway plays a key regulatory role in skeletal muscle protein synthesis^36,37^. After IGF-1 binds to the membrane receptor IGFR1, it promotes protein synthesis by activating Akt-mTOR^38^. The Akt–mTOR pathway is also a meeting point for other signaling pathways known to promote muscle growth. For example, Wnt7a can participates in this pathway to induce muscle fiber hypertrophy by activating its receptor Fzd7^39^. Correspondingly, it has been reported that 5′-adenosine monophosphate-activated protein kinase (AMPK) can inhibit the activity of mTOR complex 1 (mTORC1), thereby inhibiting protein synthesis^40^. In addition, the WNK1-FOXO4 axis has been reported to affect muscle fiber hypertrophy. With-no-lysine (K) (WNK) kinases, which can mediate the nuclear localization and transcriptional activity of forkhead box protein O4 (FOXO4) to promote skeletal muscle cell hypertrophy^41^. But in general, the most well-studied pathway is still the IGF-1-Akt-mTOR pathway. A large number of studies have confirmed that it is a core pathway that affects muscle fiber hypertrophy and is essential for myotube formation and muscle hypertrophy^28^.

**Figure 8:**
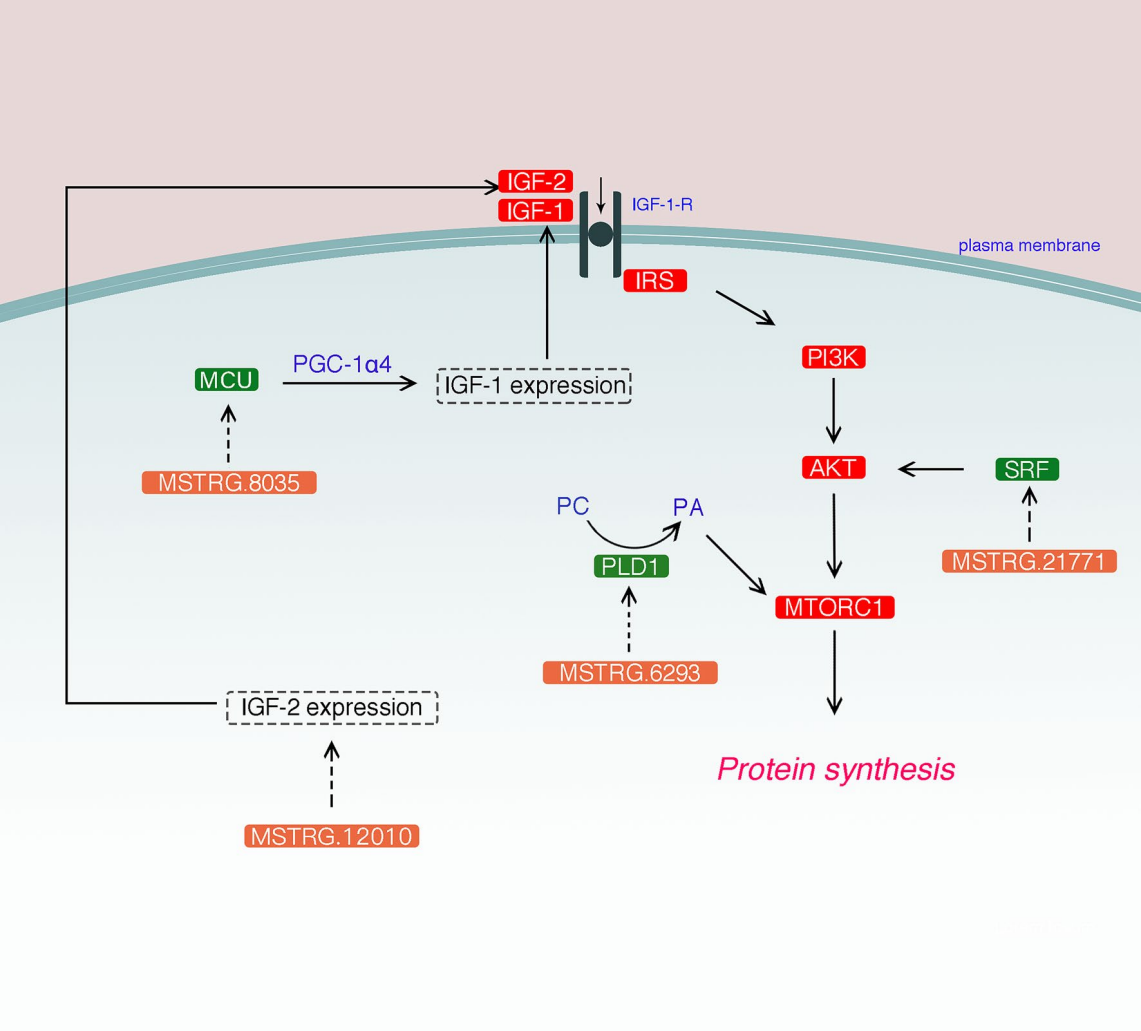
Schematic diagram of lincRNAs involvement in muscle fiber hypertrophy signaling by regulating its target genes. IGF-1, insulin-like growth factor-1; IRS, insulin receptor substrate; PI3K, phosphatidylinositol 3 kinase; mTORC1, mechanistic target of rapamycin in complex 1; IGF-2, Insulin-like growth factor 2; TNNT3, troponin T-3; SRF, Serum response factor; PLD, phospholipase D; PC, phosphatidylcholine; PA, phosphatidic acid; MCU, Mitochondrial calcium unidirectional transporter. Red indicates key genes in the IGF-1-Akt-mTOR signaling pathway; orange indicates lincRNA; and green indicates PTG.

Interestingly, we comprehensive analysis found that multiple DELs may participate in the IGF-1-Akt-mTOR signaling pathway by regulating their PTGs. Mitochondrial calcium unidirectional transporter (*MCU*) is a highly selective channel for Ca^2+^ transport into the mitochondria. Mammucari et al.^42^ reported that *MCU* participates in IGF-1-Akt-mTOR signaling by increasing Ca^2+^ level in the mitochondria, activating the PGC-1α4, which is a transcriptional coactivator; the *IGF-1* gene is activated through the PGC-1α4, leading to muscle hypertrophy^42,43^. In the present study, our analysis showed that compared with the TP group, the LW group had higher *MCU* expression level (Fig. S3), which may be associated with the growth characteristics of Yorkshire pig breeds. More importantly, we found that DEL-MSTRG.8035 is positively related to the expression of *MCU* and highly expressed in the LW group. Insulin-like growth factor 2 (*IGF-2*) is a maternal blotting growth factor that regulates prenatal skeletal muscle development^44^. It can be involved in the IGF1-Akt-mTOR signaling pathway by activating the *IGF1* receptor^45^. A significant positive correlation exists between the DEL-MSTRG.12010 and *IGF-2*, which were upregulated in the LW group. Interestingly, MSTRG.12010 was significantly negatively correlated with the troponin T-3 (*TNNT3*) gene. Wang et al*.* It is predicted that *TNNT3* can regulate muscle growth and muscle fibers^46^. *TNNT3* is an important part of pig skeletal muscle filaments, which can affect the taste and tenderness of pork^47,48^. Its expression level was low in the LW group. This may, on the other hand, find the reason for the decrease in meat quality as the growth rate increases. In addition, a potential target gene, serum response factor (*SRF*), plays an important role in controlling muscle fiber hypertrophy^49,50^. *SRF* can control the transcription of miR-486, which as a potential regulator of PI3K/Akt signal transduction in muscle cells, can phosphorylate Akt and activate the IGF-1-Akt-mTOR signaling pathway, leading to muscle fiber hypertrophy^51^. The co-expression network, suggests that DEL-MSTRG.21771 is significantly positively correlated with *SRF* expression and highly expressed in the LW group. It is speculated that MSTRG.21771 regulates the high expression of its potential target gene *SRF*, in the LW group, which possibly useful in maintaining the fast skeletal muscle rate in Yorkshire pig. *PLD1* is an isoform of phospholipase D (*PLD*)^52^, which can stimulate phosphatidylcholine (*PC*) to produce phosphatidic acid *(PA*), which can bind to mTOR and participate in the IGF-1-Akt-mTOR signaling pathway^53^. The substrate *S6K1* of *mTORC1* is phosphorylated to enhance protein translation, resulting in muscle fiber hypertrophy^54^. Furthermore, DEL-MSTRG.6293 was positively correlated with its expression, and highly expressed in the LW group. QTL results indicated that MSTRG.8035, MSTRG12010, MSTRG21771, and MSTRG.6293 were all mapped to the QTL loci, such as Loin weight QTL, Loin muscle area QTL, and backfat above muscle dorsi QTL, which are related to muscle development. It is speculated that these DELs may be related to skeletal muscle development, and participate in the IGF-1-Akt-mTOR signaling pathway by regulating the expression of its potential target genes, thereby affecting the muscle fibrous hypertrophy process. However, the specific molecular regulation of this phenomenon remains unclear, and further studies are needed. We speculate that DEL may affect muscle protein synthesis by regulating its PTG to participate in the IGF-1-Akt-mTOR pathway (Fig. 8).

In conclusion, based on our analysis of this data. We identified linRNAs that may cause differences in the growth of skeletal muscles of Tibetan and Yorkshire pigs, and discovered a number of novel linRNAs. In addition, we speculate that multiple DELs may participate in the IGF-1-Akt-mTOR pathway by regulating their potential target genes, ultimately affect muscle protein synthesis and the size of muscle fiber. Our findings provide valuable references and new ideas for lincRNA research.

## Methods

### Datasets used in this study

In this study, we obtained previously published RNA-seq data representing six transcriptomes from the GEO database (ID: GSE99749). Two sows (Tibetan pigs (TP) & Yorkshire pigs (LW)) used for RNA-seq were raised in the Tibet Agricultural and Animal Husbandry College Farm, under the same dietary and drinking water standards. Animal care and all experimentation were conducted in accordance with the guidelines pre-approved by the Tibet Agricultural and Animal Husbandry College Farm Institutional Animal Care and Use Committee. All samples were taken from longissimus dorsi muscle tissue of the embryo as described by Shang et al^10^. Divided into two groups (Tibetan pigs (TP) & Yorkshire pigs (LW)) according to breed. After 60 days of fertilization, each group randomly selected a pregnant sow and randomly took out nine embryo samples. The nine embryonic samples were randomly divided into three parts, each part as a biological replicate (each RNA library contains an equimolar ratio of RNA from the three samples.), and each group contains three biological replicates. The pig gene annotations were downloaded from ftp://ftp.ensembl.org/pub/release-91/gtf/sus_scrofa, and the non-redundant reference sequence database was downloaded from https://ftp.ncbi.nih. gov/blast/db/.

### Ethics statement

Animals care and all the experimentation in this study were carried out in accordance with the pre-approved guidelines from Regulation Proclamation No. 5 of the Standing Committee of Hubei People’s Congress. All experimental protocols were approved by the Ethics Committee of Huazhong Agricultural University, Wuhan City, Hubei Province, P. R. China.

### Animals and sample collection

The experimental Yorkshire pigs were provided by the National Livestock Engineering Research Center of Huazhong Agricultural University. All Yorkshire pigs were raised under the same temperature, humidity, ventilation conditions and feeding standards. After fasting for 12 h, three sows 55 days of gestation were randomly selected and euthanized by electric shock and rapid bleeding. Then, we collected 3 embryos from each sow for a total of 9 embryos. Collecting the longissimus dorsi muscle of the embryo and stored them in liquid nitrogen for later use.

### RNA-Seq reads mapping and initial assembly

We use Fastqc to evaluate the quality of sequencing reads in the data. Low quality reads were removed using Trimmomatic (version 0.3.2)^55^ with the default parameters. The clean data obtained were aligned to the Sus scrofa genome (SusScrofa11.1) from University of California Santa Cruz (UCSC) using the Hisat2 (version 2.0.1) default parameters. Sorting mapped reads and remove duplicates via Samtools(version 0.1.19)^56^. In addition, we assembled the read map using the default parameters of Stringtie (version 1.2.2)^57^. At the same time, we set the Stringtie's "-G" option for the novel transcript assembly. Finally, we used the merge function of Stringtie software to combine the transcription files from six samples (GTF format) into a non-redundant transcriptome file.

### Pipeline for lincRNA identification

361 lincRNAs were screened based on established pipelines in our laboratory^8^. The complete pipeline follows the one shown in Fig. 1A. Step 1, Transcripts representing intergenic transcripts classified as "U" were screened using the gffcompare program Stringtie (version1.2.2). Step 2, based on the transcript characteristics of lincRNA, transcripts with transcript lengths greater than 200 bp and exon numbers greater than 2 were screened. Step 3, the coding potential of lincRNAs were verified using a coding potential calculator (Cpc) tool^58^, and the lincRNAs with Cpc value < 0 was entered into the downstream screening program. Step 4, to improve the accuracy of lincRNA screening, we predicted the coding potential of transcripts from multiple perspectives. We translated the transcript into six possible protein sequences, which were then transcribed and compared to the Pfam database. Finally, no Pfam hit (E value < 1e−5) transcripts are retained. Step 5, Transcripts were aligned with NCBI NR and UniRef90 databases using the Blastx program, and transcripts of similar proteins in known proteins were filtered (E value < 1e-5). Step 6, to minimize the chance of false positives, transcripts that were not expressed (FPKM values) in all samples were removed.

### Comparison of characteristics between protein- coding gene and lincRNA

We selected 45,788 "gene_biotype = protein_coding" transcripts from the pig's genome annotation file (SusScrofa 11.1) to define them as transcripts of protein coding genes. In addition, we use the "blastn" instruction to divide lincRNA into known lincRNA and novel lincRNA. We then identified and compared the transcript lengths, exon lengths, exon numbers, and FPKM averages for these three categories.

### Differential expression analysis of lincRNA

We used the counting software Htseq^59^ to count the number of reads in the six samples, and then divided the six samples into two groups according to the variety, namely TP and LW, and compared them in R using the "Deseq2" package^60^. The gene of |log2FoldChange|> 1, *padj* < 0.05 is a differentially expressed gene. Then, it is calculated by taking the intersection of the potential lincRNA obtained from the pipeline to obtain the differentially expressed lincRNA (DELincRNA), at the same time, it is intersected with the protein coding gene expressed in the sample to obtain a differentially expressed protein encoding gene.

### lincRNA target gene prediction

Consistent with previous research. Because lincRNA can regulate its potential target genes in cis. Based on this, we used Bedtools software (version 2.17.0) to search protein-coding gene in the 100 kb upstream and downstream of the lincRNA locus, and used R to calculate the Pearson correlation coefficient between DEL and protein-coding genes. Finally, protein-coding gene with a correlation coefficient greater than 0.9 were identified as potential target genes for DEL. At the same time, if the protein-coding gene is differentially expressed in the two groups, this protein-coding gene is a potential target gene for differential expression.

### Function enrichment analysis

Due to the limitations of pig genome annotation, this study included background human orthologous genes^61^. After transforming the pig gene into a human gene in the Ensembl website, gene ontology (GO) and the Kyoto Gene and Genomic Encyclopedia (KEGG)^18–20^ pathway enrichment analysis were performed in Metascape^21^. Subsequent selection of *p* value less than 0.05 is a valid result.

### Prediction of DELs function by QTL

In this study, the pig QTL annotation file was downloaded from the animal QTL database (https://www.animalgenome.org/cgi-bin/QTLdb/SS/index), and the location information of DEL was proposed in the non-redundant transcription file according to the ID of DEL. After that, we performed QTL mapping on DEL using the BedTools (version 2.17.0).

### Correlation verification between DEL and its PTG

We used the longissimus dorsi muscle from nine 55-day-old embryos of Yorkshire pigs and performed RT-qPCR to verify the expression correlation between DELs and PTGs. For quantitative verification, in the first step, total RNA was extracted using Trizol reagent (Invitrogen, Life Technologies, CA, USA) and performed according to the manufacturer's instructions. To prevent the degradation of RNA, before cDNA synthesis, we measured the purity and concentration of total RNA at 260 and 280 nm with a microphotometer (Thermo, NanoDrop 2000, United States). At the same time, we conduct a gel electrophoresis test to detect whether the RNA is degraded. There are usually three frequency bands, of which 28S and 18S are clear, and the brightness ratio is about 2:1, indicating that there is no degradation. Next, cDNA synthesis was performed using the RevertAid First Strand cDNA Synthesis Kit (Thermo, Wuhan, Cat#k1622).According to the manufacturer's instructions, qPCR for DELs and PTGs detection in Roche LightCyler 480 system (Roche, Mannheinm, Germany) was performed using SYBR Green (CWBIO, Beijing, China, CW0957). Ten pairs of RT-qPCR primers were designed using the Primer 5 program (Tables S5, S6). 18S rRNA is used as an endogenous control gene. The RT-qPCR data were analyzed using the 2^−△△CT^ method and R scripts were used to perform related linear regression analysis.

## Supplementary Information

Supplementary Fig. S1.

Supplementary Fig. S2.

Supplementary Table S1.

Supplementary Table S2.

Supplementary Table S3.

Supplementary Table S4.

Supplementary Fig. S3.

Supplementary Table S5.

Supplementary Table S6.

Supplementary Information.

## Data Availability

The sequence datasets of this paper from the GEO database. The GEO data set ID is GSE65983 (https://www.ncbi.nlm.nih.gov/Traces/study/?acc=SRP108727&o=acc_s%3Aa).
