# Supplementary figures and images for: Transcriptome analysis reveals the long intergenic noncoding RNAs contributed to skeletal muscle differences between Yorkshire and Tibetan pig
Source: Sci Rep. 2021 Jan 29;11:2622. doi: 10.1038/s41598-021-82126-2 (PMC7846844; doi:10.1038/s41598-021-82126-2)

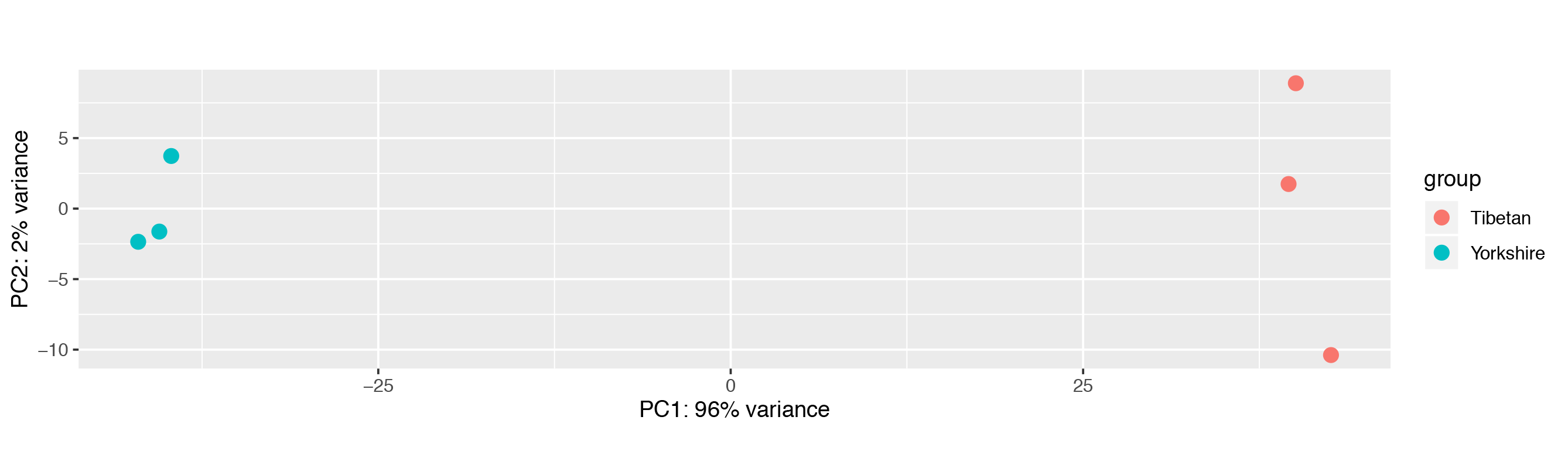

Supplement: Supplementary file 1 — Supplementary Fig. S1. [file 41598_2021_82126_MOESM1_ESM.tif]

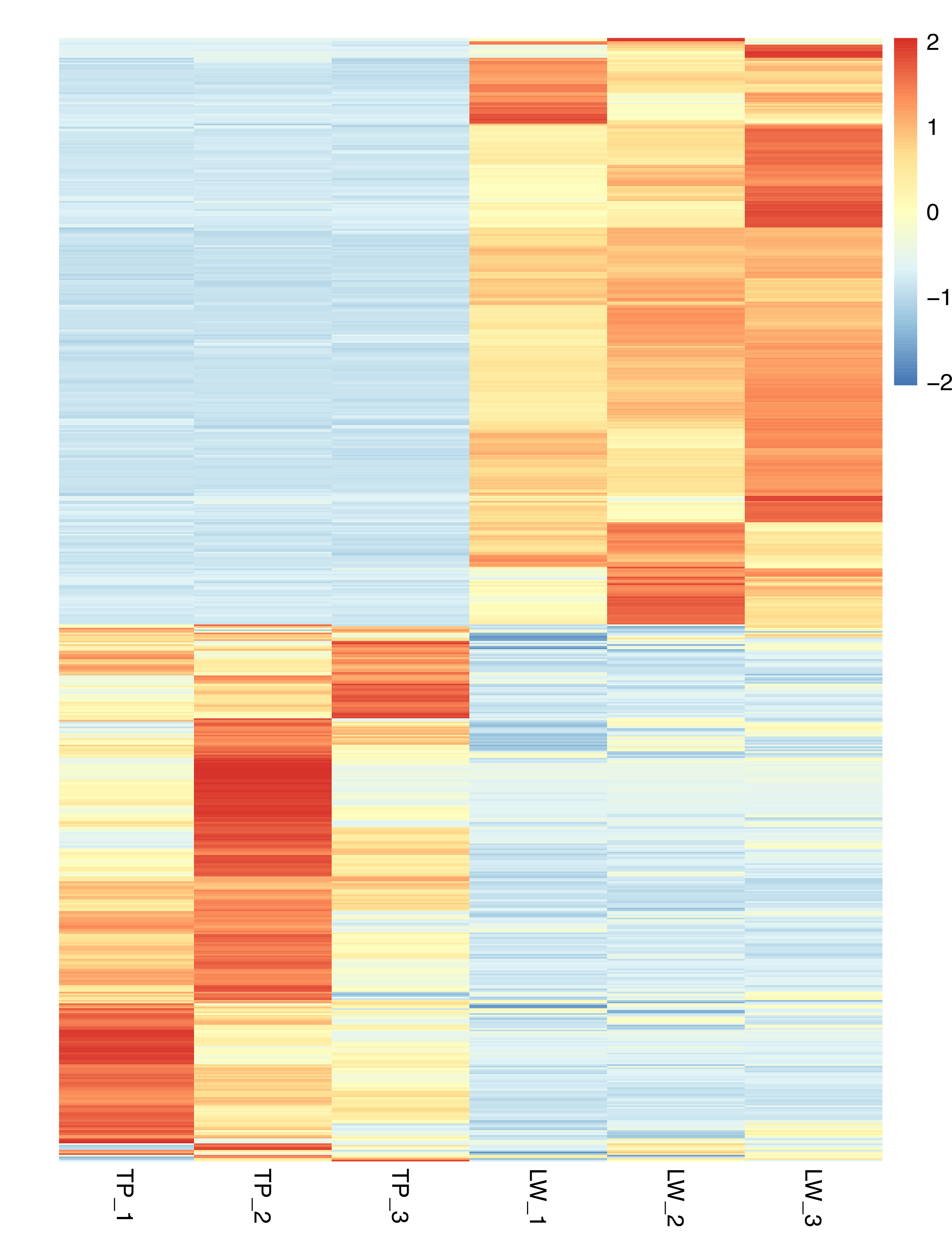

Supplement: Supplementary file 2 — Supplementary Fig. S2. [file 41598_2021_82126_MOESM2_ESM.tif]

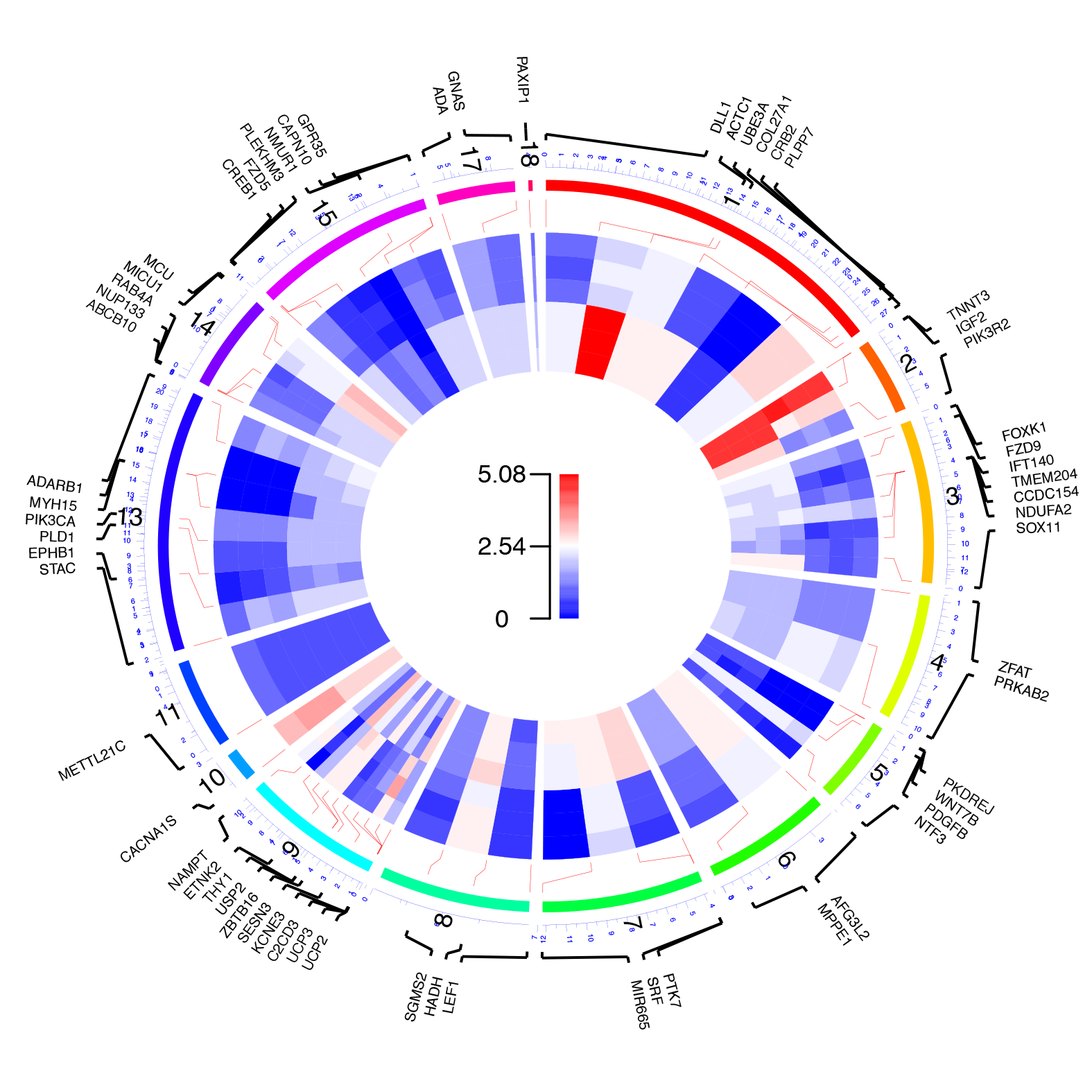

Supplement: Supplementary file 7 — Supplementary Fig. S3. [file 41598_2021_82126_MOESM7_ESM.tif]
