# Supplementary material for: Transcriptome analysis reveals the long intergenic noncoding RNAs contributed to skeletal muscle differences between Yorkshire and Tibetan pig
Source: Sci Rep. 2021 Jan 29;11:2622. doi: 10.1038/s41598-021-82126-2 (PMC7846844; doi:10.1038/s41598-021-82126-2)
Supplement: Supplementary file 9 — Supplementary Table S6. [file 41598_2021_82126_MOESM9_ESM.docx]

**Table.S6. Sequence information of lincRNAs used in RT-qPCR.**

**MSTRG.14875:**

AGTTTTATTTCTGGCAAAATGAGACGGGTGAGGAAGTGCCCCAAAGAGGCATCAGGAAGGTCAGTGCATATGTGGTCAGTGCATATATTCTTGGCAGGAGGTCGCGGCTTGTCTGGAGGCCCAGTTTGCTCCTCTGGATATGAGGAGATGAAAGAAACTGGACTCATAAAATCTCCGGAAAACACCCAACTCTCTGAAGGCCCGTTCTGCCAGTTTTTCCCAGAGCACAGGGTGCCCTGTTCCTGATCTCCGCCCCGAACTGCTCTCAGGGGCGCTGAAGTTCAGCTACCTCATTCTTGCAGAGCCAGATGACGAGGGAGCCTGTTTAGTTGCAGCTGTAACGTGCAGCCGCCCTGGTCAGCACCTCTGCCTCTGGAGGATCTCCTGCGCCTGCTTTGTGGCGGGAGAAGCACCAGCCATGGGCACTATTCTTCGCCCCCCAGAAACTCTAGAGACAGGTGAGCAAAGGTGGCAGGCTGGAGGGTTCCCAGCTCAGCAACATCTGGGAGACCTCCCTGCTCCACCCCGGATGCCCGGCGGAGGCTCCGATCCAAACCCAGAACTGTGTTGAGCTGCTGTGTCAACAGAGGCACCTGTGTGCCCATATCCTCCTTTCTCACTGGGCCCCACCCCACCTTTTCCATGCCAGAACCCCACCCAGAATCACGGGCAAACCAGTTCAGCCACCGGGAACCCCAGGAGCACCTGCTGTGGGTGGGAGGAGGGGGTGCTAAGTGTGGGGAGAATGGGCCACCATCACTCTGGTGTGCAGTCTGTAGGGCTTCCTGGAGGAGGTGGAGATCAAAGAGCCCTGAGGTTAGGGGTGGAGCTGCCGACTGCAGCTAATGCAGCCACTTGGTTGGTATGCTCCAGGGCATCTGGGAGCCACCCTGGGACAGCAGAGTGGGTGCAGAGCTGGGGGTCAAGGAGAGTGGCTGGGACTTCCATCCTGGCCAGGACAGCCTCCAAGGGCACCTAAACCTGTCCCAGTCTTGATGACTGCCTTGGGTGGGTGACAGAAACAGGACGTCCTGGAGGGCATCTCTAGAGCCAGGCCGTCCTTGGCTGTGAAATAATGGGCGTTGTGTGCTGGGGGCTGCTTCCAGGCCAGCTGCTATAGCTGGGAAGCCGTTGGCAGGAACCTGGGACAGCATGAAGACTGAGGGGGTGGCGTGGAAATGTGTTTCCATCCAGAGGCAGTGGCGGATGCCCTCCGCCCATGGGGTCCCCAGGGCTGCGCCGGGGCCAGAGCCTGGGAGGAGCACGCAGACCCACAAGAGGGAATGAGCACATCCACATGTCCCCCTCTGTCCCCAGCTCCTGCTGCCACTGGTCCCCATTCTAGGTGAAGGCCCGATGCCAGGACCTGGATCCTGAGGCATCTGAGAGGACAGCAGGCGCTGGTGTCCGGGAGCTGGCCTGCTGTTCTCCCGTGGGGCGTCATCCATCTCAGGGAACAGCAGTGTGGGACCAGAGACCATAGGCTGGTCTCTGTGACCATGACAGCTCAAGACAAAACACCACCTTCCATAATCGCATCTGACACGGCCCAAACCTCAACAAAGACCAAGTACCCCATCCTGGCTGAGGGGAGGGACCCTGGTCCTTTACCAGCCACAGCTGGAGCTTCCCTTTTTCTTGCCTCCTTCTGGATAAGATGTGTTAGGATGCTGCACTGGGTTGAACGGTGTTCCCTAACTTCACGTCCCGCTGGAGCCTCAGCCCGTGACCCCATTGGCATCTAAGTTCTGCGGCTGTAACGGGTTGAAGCGATGTCATGCTCGATGGGGCGGGCCCTAAGCCCAGGGCCTAGAGTCCTTATGAGAAGAGAAAAGGCCCGGAGACACGCAGCAGAGGGGCAATGTGACGAGGCAGCAGAGACTGGAGCGACGCAGCCGCAGGCTGAGGGCCGGCGGGGGCAGCCGGCTTCGCCGGGAGCCGGGAGAAAGGCCAGGAGCAGACTCTCCTCTCCTGAGCTGGAGGAGGCAGCGTTGTTTTAAACCACCCATTAGGGGTCCTTCATTACAGCAGCCCCAGGGGAACGACAGACGCCCAACCCAAAGCAGAGCCCTCACCCAACACCCGCAGAACCGCAGCCCTGTCTTCTCTCTCTTTTTTTTTTTTTTTTTGTTTTTCTTTTGTTTTCTTTAGGGCTGTACCCGTGGCACATGGAGGTTCCCAGGCCTGGGGCTGAGTCGGAGCTGTAGCTGCTGGCCTCCACCACAGCCACAGGAACATGGGATCCGAGCTACGTCTGCAACCTACACCACAGCTCACGGCAACACCAGATCCTTAACCCACTGAGCGAAGCCGGGGATCAAACCCTCGCCCTCCCGGATGCTAGTCGTGTTTGTTAACCACTGAGCCATGACGGGACCTCCACCAACCAGAGCCCTTTCTACCGTTCGCCTGCTGAGACCCGTGCTGAGATTCCGAGCTGTGGTTCCCCTGTGCTTAAACCCAGCTGTCCAGTGACACAGGCGGTGGCGGCGGCTGACCAGAGGCGACGACAGCCCCAAATGCTGTGGTCTCTTGACTCTCCCACGCGGCGGGGCCTAGAGGGGGAGAAGCCCCAACCAGATGTGTCCGGGGCTGGCTGGGCGCTGGGTGACAGGTGCCCAGCTCCGCTGGCGATGCATGCTCCTGTCCAAGGCCCCCCCCCGCCCCCAGGCATAAACCAACACCTCAGCCCTCATTATCTCAAAGCACACAGAGAAACCTCACACAAGCTCTAATGTCCCGCTAATGGGAGGGAGG

**MSTRG.2530**

AGGACTGGGAGTGGATCTCTGTGCCCAGGAGGCCTCATCGCCTTGGGTCAGGTGTCCTGGAGCAGGAGACGGTGACCACCAAAGGTGACCAAGCCAGCTCAGGATGAAGAAGAGTATCTGCATCCACCTGCTGCCTGGCCCCAAGCACAGCCCGTTTCATCTCTGTTGATACACCCCATCACCGCTCCCATAGTTCTCACAGGCAGCCATCCTCTTCTCACCACGCCTACCCCAGTCCGGCCTCCCTGCGGGCCTTTTCCAAACCTCTCTGAAGCAGAAAGGACTTCGAAACCCCCAGCGCTGACCCGTCATTCCCCAAGTCGAGACGCAGAGCTGGCTCCCCATTGCCCTGAGTTCCAACTCCATCTCTGCCCCGGGGCATTTGCCAGACAGGCCGCCCACCCCCGCTGTAGCCTAGCACCACCCCCAAACGGTGCAACCAGCTTCCAGCCCCCAGTCGAAAGGACTTCCTGACCCTGGAACTCAAAAGTCAAACCAGGTTCCCAGGTAAAATGGTCTTGCATTCCCACCGCTCTCTGCCCCACCCAGGCTGAGCCTTCTAGTGAAAAGAAGCTCTTACAACTCTGTTGAGTATGTTTGCACATCGCTAGTCATAGCTAACCTCCCGAAACAAAGCAACATGGCAGGTAAAGCAATTTAACAAAAGCAGATGCTTTTCTTTATTGTCTGAGTTTTAAATCCTTTTATA

**MSTRG.12010:**

CAGATTCCGGTAACTGAGTTTATTGATGAGTCCAGGGCTCTGGGGTGGCCCTGGCGAGGAGGGGCACATGACGAGATGGACACTCAGTGCAGCGTTTGAGGTCTGCTCGGTGGTCGGAGCTTCCAGACTAGGAGAGGGGAGGGCGGGGCCCCACACACTCAGGCCCCCACCCAGTCACCCCCAGACAGAGATGGACAATGGCAGGTGCTGAGGGGGCGCGGGCCGAGGAGGCCAGGCCACTCGGCAGCTCACACACCCGCTCCGAGACCCGGGGCTGGTTCCAGGACTCCAAACCAGTGGAGTGGCCGGGCGGGGGGTGGGGAACCCCGCGGGTGCCGTTGGTCCGGTGGTGTGGGACGTCTGTTCCTTTGGCTCAACCTGCGGACGAGGACAGAG

**MSTRG.12010:**

CAGATTCCGGTAACTGAGTTTATTGATGAGTCCAGGGCTCTGGGGTGGCCCTGGCGAGGAGGGGCACATGACGAGATGGACACTCAGTGCAGCGTTTGAGGTCTGCTCGGTGGTCGGAGCTTCCAGACTAGGAGAGGGGAGGGCGGGGCCCCACACACTCAGGCCCCCACCCAGTCACCCCCAGACAGAGATGGACAATGGCAGGTGCTGAGGGGGCGCGGGCCGAGGAGGCCAGGCCACTCGGCAGCTCACACACCCGCTCCGAGACCCGGGGCTGGTTCCAGGACTCCAAACCAGTGGAGTGGCCGGGCGGGGGGTGGGGAACCCCGCGGGTGCCGTTGGTCCGGTGGTGTGGGACGTCTGTTCCTTTGGCTCAACCTGCGGACGAGGACAGAG

**MSTRG.21771:**

CTGAGAACTGAGCCTCTGGTTGGCTTGGGCAGGGCCAGAGTTAGGTCAAGAACAGAGGTCCAATCAATGTCATTTAGGGATGCGGGAAGGGTGGAGAGATTCAGAGAAAGCAGAAATCCAGCACGTGGAGGACCTTCCAGTTTATCCTCGCACTGCCCCTCGGCACCCAGGTCAGACATCCCCTCCAGGCAAGAAAGAGTGTCGGGACCAGATTCACCCTCCCCTGTATTTTGTTTGGTTGGTTAGTTTTCACAGGTGACTTTCCTTTCTCAGATATCTCAGATACGGTGTGGAGGAAACGGGGCTGAGCATCTCTGCCCTAACAGACACGATACTCACCTGACTCGGGTACTTTCTTAGTCTCGTTTCCTCCCCATTCCAGTTGCTGGTGACATCCTTTAGGAGGCCAATTTTTTGAAAGTGGGAAATGTCTGGAGTTCTAGTCTTTTT
