# Supplementary material for: Transcriptome analysis reveals the long intergenic noncoding RNAs contributed to skeletal muscle differences between Yorkshire and Tibetan pig
Source: Sci Rep. 2021 Jan 29;11:2622. doi: 10.1038/s41598-021-82126-2 (PMC7846844; doi:10.1038/s41598-021-82126-2)
Supplement: Supplementary file 10 — Supplementary Information. [file 41598_2021_82126_MOESM10_ESM.docx]

**Supplementary file information**

**
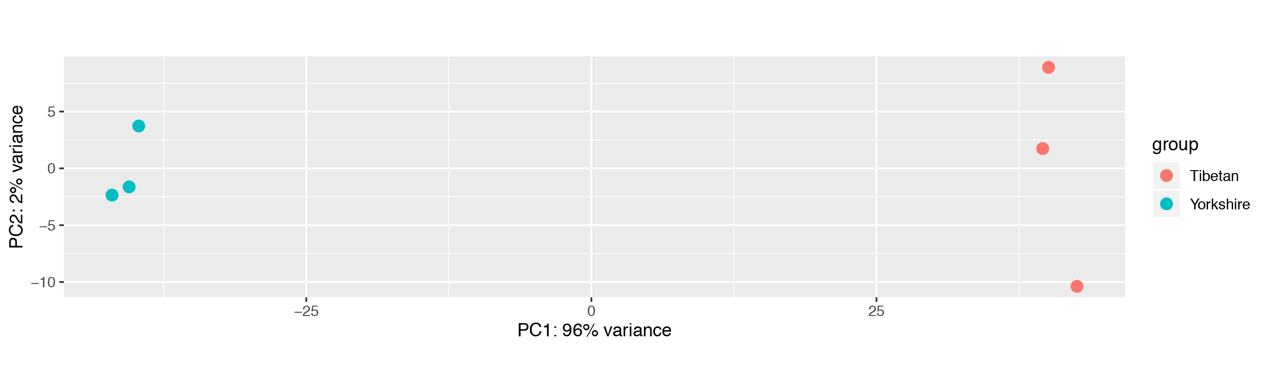
**

**Supplementary file 1: Fig.S1.** PCA analysis results of RNA-seq data.


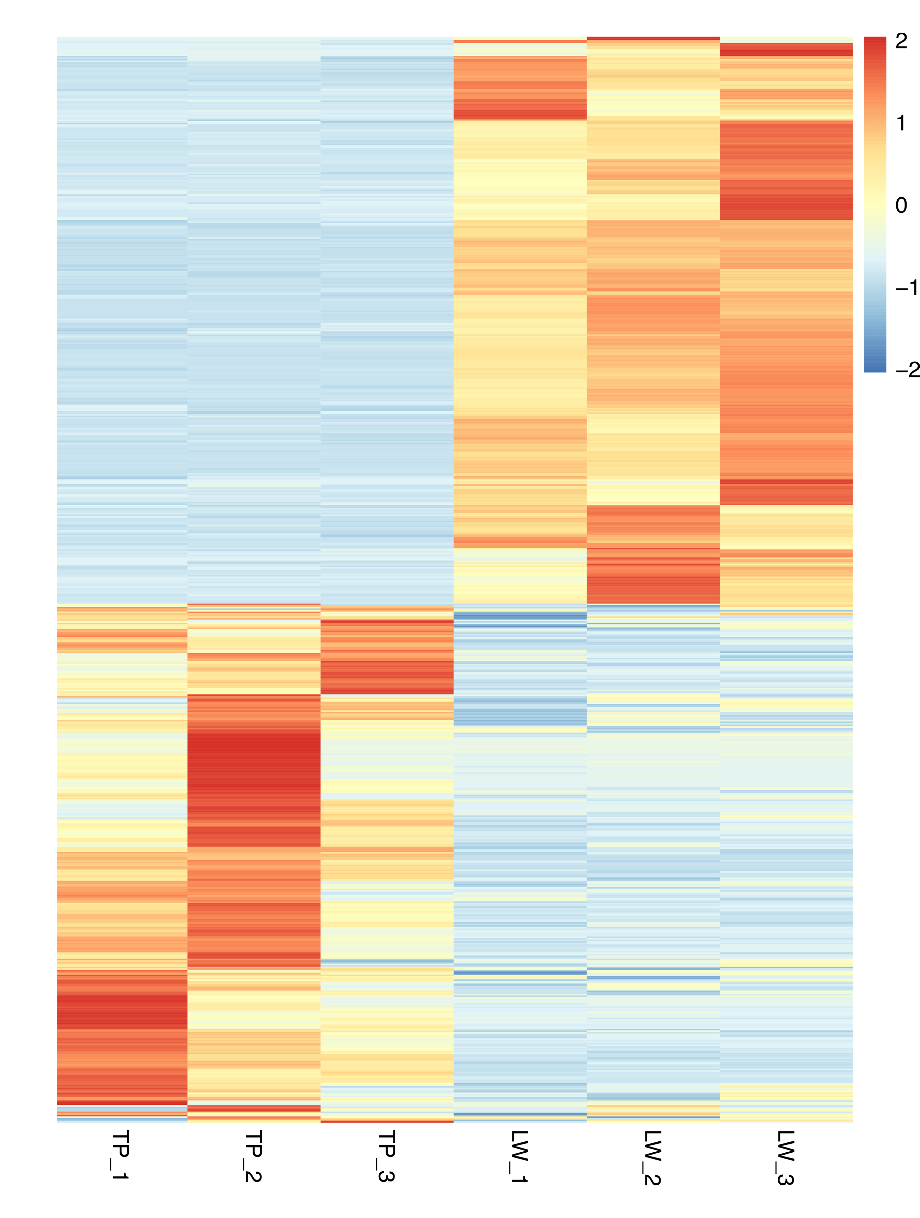


**Supplementary file 2: Fig.S2.** Differential expression analysis of differential protein coding genes. the bar code represents the color scale of the log_10_^(FPKM)^ .

**Supplementary file 3: Table.S1.** The statistics table of DELs target gene.

**Supplementary file 4: Table.S2.** Gene ontology and pathway analysis of adjacent genes of lincRNAs.

**Supplementary file 5: Table.S3.** Statistical table for QTLs analysis of DELs.

**Supplementary file 6: Table.S4.** The expression regulation relationship between DEL genes and their PTGs.

**
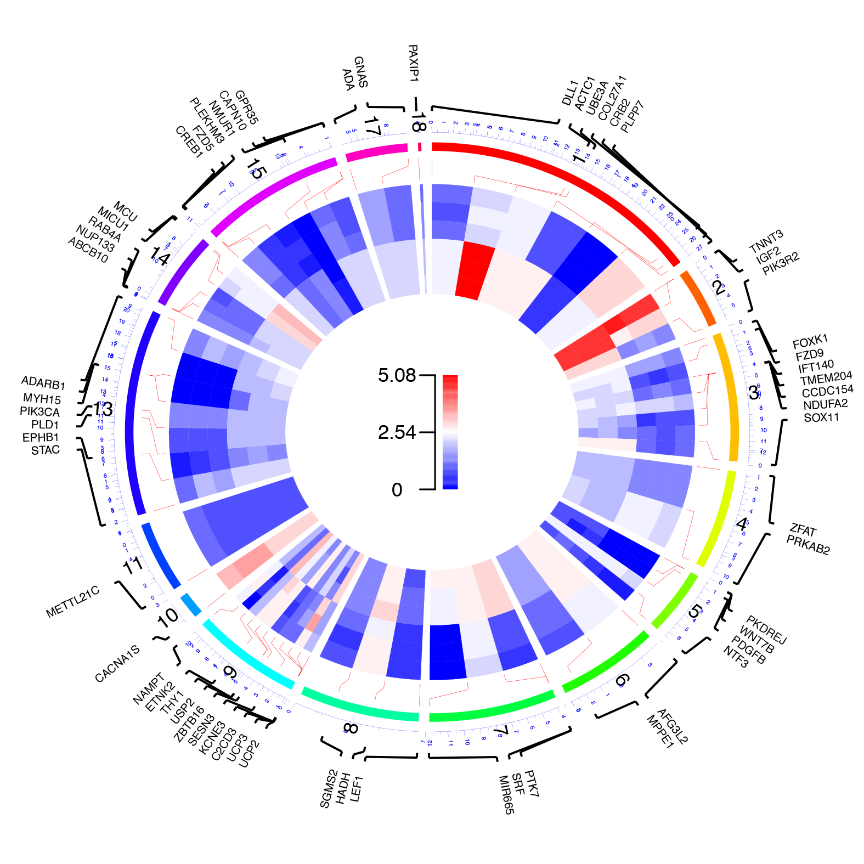
**

**Supplementary file 7: Fig.S3.** Heatmap of muscle development-related differentially expressed genes. the bar code represents the color scale of the log_10_^(FPKM)^.

**Supplementary file 8: Table.S5.** The information of ten pairs of RT-qPCR primers.

**Supplementary file 9: Table.S6.** Sequence information of lincRNAs used in RT-qPCR.
